# Crystal packing analysis of *in situ* cryocrystallized 2,2,2-tri­fluoro­aceto­phenone

**DOI:** 10.1107/S2056989017016590

**Published:** 2018-04-17

**Authors:** Dhananjay Dey, Abhishek Sirohiwal, Deepak Chopra

**Affiliations:** a Crystallography and Crystal Chemistry Laboratory, Department of Chemistry, Indian Institute of Science Education and Research Bhopal, Bhopal 462066, Madhya Pradesh, India

**Keywords:** crystal structure, *in situ* cryocrystallization, fluorine-based inter­actions, inter­molecular inter­action energies, Hirshfeld surface analysis

## Abstract

Crystals of the liquid compound 2,2,2-tri­fluoro­aceto­phenone (TFAP, C_8_H_5_F_3_O) were obtained using the state-of-art *in situ* cryocrystallization technique. TFAP crystallizes in the monoclinic space group *C*2/*c*, and its crystal structure is mainly stabilized by a set of C—H⋯F, C—H⋯O, F⋯F and F⋯O supra­molecular contacts. The overall mol­ecular arrangement shows the formation of mol­ecular sheets parallel to the *bc* plane, which are in turn stacked along the *a*-axis direction. The weak inter­actions have been studied thoroughly, performing both a Hirshfeld surface analysis and theoretical calculations, to obtain the inter­molecular inter­action energies. A structural comparison of this compound with the previously reported substituted analogs was also carried out, showing a qualitative difference in terms of packing behaviour.

## Abstract 2

In this present study, the crystal structure of 2,2,2-tri­fluoro­aceto­phenone (TFAP) is determined using *in situ* cryocrystallization techniques. The main objective of this work is to study its crystal packing associated with the various inter­molecular inter­actions, along with a detailed comparison with the features of substituted analogs. It is inter­esting to note how the chemical substitution of different functional groups influences the crystal packing, the electronic environment of the mol­ecule and the nature of the various inter­molecular inter­actions.

## Chemical context   

The use of green, efficient, metal-free and inexpensive catalysts is the desire of every synthetic laboratory. The importance of metal-free catalysts is well known among synthetic chemists. In this class of catalysts, 2,2,2-tri­fluoro­aceto­phenone (TFAP) is well known, because it is cheap and commercially available.
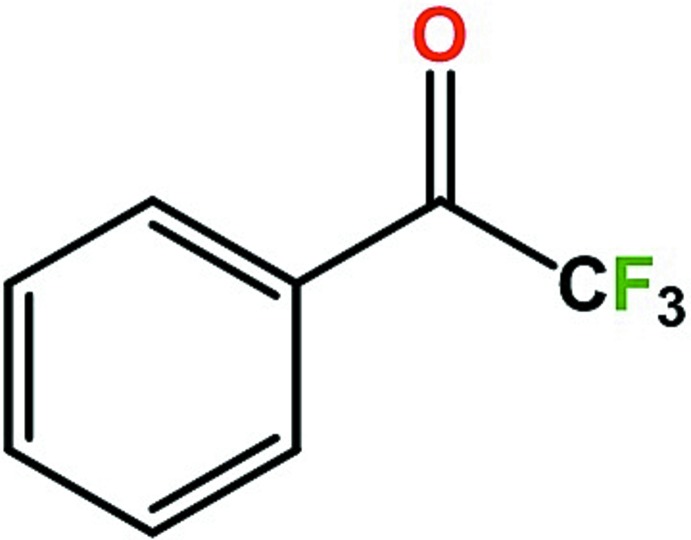


Research work in recent years has shown that TFAP can be used as a green organocatalyst in synthetic procedures, *e.g.* for the epoxidation of alkenes (Limnios & Kokotos, 2014*a* ▸), the oxidation of allyl­oximes to form isoxazoline (Triandafillidi & Kokotos, 2017 ▸), the oxidation of aliphatic tertiary amines and azines (Limnios & Kokotos, 2014*b* ▸) and for the synthesis of substituted tetra-hydro­furans (Theodorou & Kokotos, 2017*a* ▸), indolines and pyrrolidines (Theodorou & Kokotos, 2017*b* ▸), besides being used for the synthesis of fluorinated polymers (Guzmán-Gutiérrez *et al.*, 2008 ▸). Inter­estingly, TFAP has been also used for probing inter­molecular inter­actions involved in the bi-mol­ecular complexes formed on Pt(111) surfaces (Goubert *et al.*, 2011 ▸). In fact, TFAP is also an excellent example to study the enanti­oselective hydrogenation on Pt surfaces (Cakl *et al.*, 2011 ▸). Keeping in mind both the important applications of this mol­ecule and our work on inter­molecular inter­actions involving organic fluorine, we decided to determine the crystal structure of this compound. It is worth noting that since TFAP is a liquid at room temperature, a crystal structure determination using conventional methods is not feasible; hence, this class of compounds needs special experimental settings. The method for obtaining crystals of these compounds is called the *in situ* cryocrystallization technique (Boese *et al.*, 2003 ▸; Choudhury *et al.*, 2005 ▸). In the recent past, we have employed this technique to obtain crystal structures of both organic (Dey *et al.*, 2016*a*, ▸ *b* ▸) and organometallic liquids (Sirohiwal *et al.*, 2017*a* ▸). We believe that this study delineates the importance of fluorine-based inter­actions, in addition to other weak inter­actions, which play a role in the crystal packing of TFAP.

## Computational methodology   

All the calculations were performed at the crystal geometry, where hydrogen-atom positions are fixed to their respective neutron values (Allen, 1986 ▸). The lattice and inter­molecular inter­action energies were computed using the PIXELC module of the *CLP* program (Version 12.5.2014; Gavezzotti, 2003 ▸, 2011 ▸), which partitions the total energy into Coulombic, polarization, dispersion and repulsion energies. For the same purpose, the mol­ecular electron density was computed at the MP2/6-31G (d, p) level of theory using *Gaussian09* (Frisch *et al.*, 2009 ▸).

## Structural commentary and supra­molecular features   

**Figure 1:**
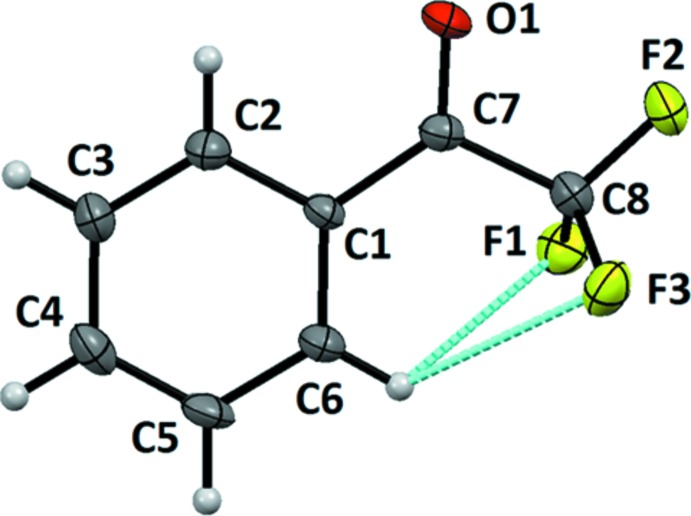
Displacement ellipsoid plot of TFAP drawn at the 50% probability level. Weak intra­molecular inter­actions are shown as cyan dotted lines.

The single-crystal X-ray diffraction analysis reveals that the title compound crystallizes in the space group *C*2/*c*, and confirms the presence of one –COCF_3_ functional group attached to the phenyl ring (see Fig. 1 ▸). The backbone of the mol­ecule formed by the atoms O1/C1–C8 is essentially planar, with a maximum deviation from the plane of 0.053 (1) Å for C8. In the mol­ecule, two intra­molecular C—H⋯F inter­actions are present, involving C6—H6 and the atoms F1 and F3 (C6—H6⋯F1, 2.48 Å and 115°; C6—H6⋯F3, 2.55 Å and 116°; Table 1 ▸). A total of seven mol­ecular pairs are extracted from the crystal packing based on their stabilizing contribution towards the total lattice energy. Their detailed energy decomposition analysis is listed in Table 2 ▸. These mol­ecular pairs are associated through various inter­molecular inter­actions involving aromatic C—H groups as donors and C—F and C=O moieties as acceptors. The crystal packing is further stabilized by the presence of π–π stacking and of different types of atom–atom contacts, such as inter­molecular F⋯F, F⋯O, and H⋯H contacts.

**Figure 2:**
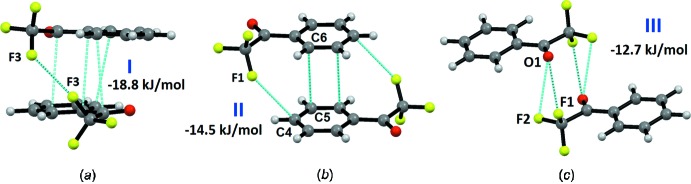
Mol­ecular pairs (*a*) I, (*b*) II, and (*c*) III with their stabilization energies.

The strongest mol­ecular pair I (Fig. 2 ▸ *a*), with an inter­action energy of −18.8 kJ mol^−1^, is formed *via* mol­ecular stacking inter­actions and inter­molecular type I F⋯F contacts [F3⋯F3, 2.8743 (1) Å and C8—F3⋯F3 139°]. In this case, the dispersion contribution (78%) is more significant in comparison to the electrostatic contribution towards the total stabil­ization of the dimer. The centrosymmetric mol­ecular pair II (Fig. 2 ▸ *b*), which is also formed due to π–π stacking, and to inter­molecular F1⋯C4 inter­actions, shows an inter­action energy of −14.5 kJ mol^−1^ (18% electrostatic and 82% dispersion contribution). Motif III (involving O1 with F1 and F2), with an inter­action energy of −12.7 kJ mol^−1^, is stabilized *via* inter­molecular bifurcated F⋯O inter­actions with indiv­idual distances of 3.1436 (1) and 3.0457 Å (Fig. 2 ▸ *c*). This shows how inter­molecular F⋯O contacts provide a significant contribution towards the stabilization of the crystal packing, as already investigated in our recent study in terms of the associated nature and energetics (Sirohiwal *et al.*, 2017*b* ▸).

**Figure 3:**
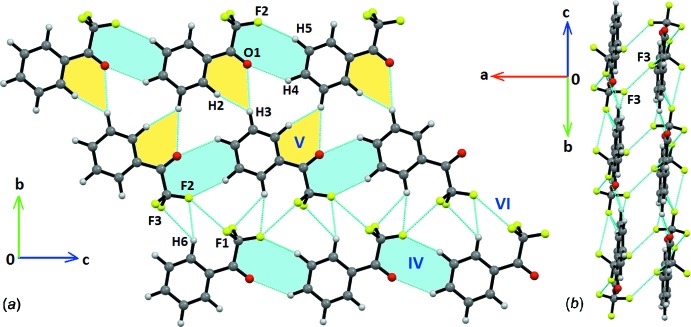
Packing network of TFAP showing (*a*) the mol­ecular sheet formed *via* weak inter­actions in the *bc* plane and (*b*) the mol­ecular stacking of two parallel sheets. Weak inter­actions are shown as cyan dotted lines.

The overall mol­ecular arrangement shows the formation of a mol­ecular sheet parallel to the *bc* plane (Fig. 3 ▸ *a*). This sheet is constructed *via* the mol­ecular pairs IV (−10.0 kJ mol^−1^), V (−6.9 KJ mol^−1^) and VI (−6.0 kJ mol^−1^). It is inter­esting to note the dominance of the electrostatic (54%) over the dispersion (46%) contribution in case of motif IV, which is not to be found in other motifs. A mol­ecular dimeric chain, associated with motif IV, is formed along the crystallographic *c*-axis direction, involving inter­molecular C4—H4⋯O1 and C5—H5⋯F2 inter­actions (Table 1 ▸). Such dimeric chains are inter­linked alternatively along the *b*-axis direction either *via* mol­ecular pairs V (involving C4—H4⋯O1 inter­actions and H⋯H contacts) or VI (involving bifurcated C—H⋯F inter­actions and F⋯F contacts) related by *c*-glide symmetry. Finally, these parallel mol­ecular sheets are stacked along the *a*-axis direction (Fig. 3 ▸ *b*) *via* the strongest mol­ecular pairs I. Thus, in the absence of any strong hydrogen bonds, the overall crystal packing is stabilized through weak inter­molecular inter­actions.

## Database survey   

Most of the substituted TFAPs are also liquid at room temperature and were crystallized *via in situ* cryocrystallization methods in the absence of OHCD. In particular, the crystal and mol­ecular structures of 4-fluoro TFAP (SIDMAU), 4-chloro TFAP (SIDLUN), 4-bromo TFAP (SIDLOH), 3-bromo TFAP (SIDLEX), and 3-nitro TFAP (SIDLIB) have been obtained and reported (Chopra *et al.*, 2007 ▸).

**Figure 4:**
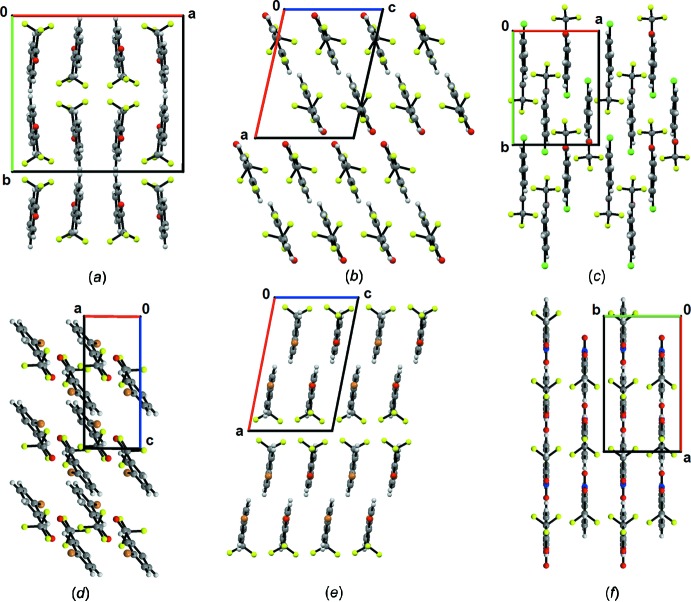
Mol­ecular assembly in (*a*) TFAP and substituted TFAPs: (*b*) 4-fluoro TFAP, (*c*) 4-chloro TFAP, (*d*) 4-bromo TFAP, (*e*) 3-bromo TFAP and (*f*) 3-nitro TFAP.

Fig. 4 ▸ highlights the similarities and differences of the mol­ecular assemblies for these structures in comparison to unsubstituted TFAP. Inter­estingly, in most of the cases, the mol­ecular sheets are stacked on each other. The supra­molecular assemblies are mainly stabilized *via* various weak C—H⋯O/F/Cl/Br/N inter­actions and F⋯F, F⋯O, Br⋯O, Br⋯F contacts without the presence of any strong inter­actions. Upon substitution with F, Cl, Br and –NO_2_ groups, a mol­ecular chain associated with F⋯F contacts is observed. In particular, in the case of the *para-*substituted chloro and bromo analogs, the F⋯F chain is quite similar, wherein in the case of the *para-*substituted fluoro compound, bifurcated F⋯F contacts are present. Finally, in the case of the *m*-nitro and bromo derivatives, a centrosymmetric, dimeric F⋯F chain is observed.

## Hirshfeld surface analysis   

**Figure 5:**
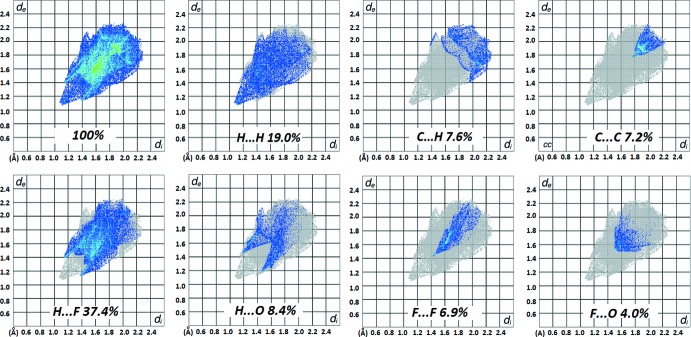
Two-dimensional fingerprint plots for TFAP, decomposed into contributions from specific atom–atom contacts.

The Hirshfeld surface analysis was performed using *CrystalExplorer3.3* (Turner *et al.*, 2017 ▸) to obtain two-dimensional fingerprint maps (Spackman *et al.*, 2002 ▸; McKinnon *et al.*, 2007 ▸), which help us to understand the crystalline environment in terms of the contributions of various inter­atomic contacts present in the crystal packing. The 2D fingerprint plots and the decomposed contributions for different atom–atom contacts in unsubstituted TFAP are shown in Fig. 5 ▸. It is observed that the contributions for H⋯F (37.4%) and H⋯H (19.0%) contacts is relatively high in comparison to the other inter­atomic contacts. Inter­estingly, in this case, the fluorine atoms present in the –CF_3_ group are more involved in the formation of C—H⋯F inter­actions rather than the formation of F··F (6.9%) contacts. The other contacts, namely C⋯H (7.6%), H⋯O (8.4%) and F⋯O (4.0%) also contribute to the overall crystal packing.

## Crystallization, data collection and structure refinement   

**Figure 6:**
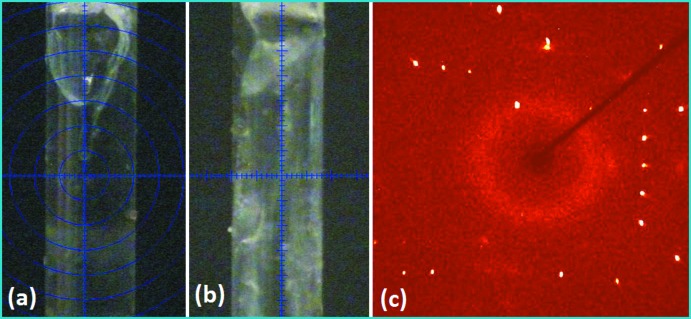
Crystal images at (*a*) 200 K, (*b*) 110 K, and (*c*) the diffraction image at 110 K.

The compound TFAP was purchased from Sigma–Aldrich and used for the *in situ* crystallization experiment without any further purification. The detailed procedure of the crystallization process is already discussed in one of our previous reports (Dey *et al.*, 2016*a* ▸). Good quality crystals (Fig. 6*a* ▸) were obtained at 200 K using a CO_2_ laser scan utilizing an OHCD apparatus. Fig. 6*b* ▸ and *c* ▸ depict the crystal at 110 (2) K inside the Lindemann glass capillary and the corresponding diffraction image, respectively. The crystal data, data collection and details on structure refinement are summarized in Table 3 ▸. All non-hydrogen atoms were refined anisotropically and the aromatic hydrogen atoms bonded to C atoms were positioned geometrically and refined using a riding model with *U*_iso_(H) =1.2*U*_eq_(C) and C—H distances of 0.95 Å.

## Supplementary Material

Crystal structure: contains datablock(s) I, global. DOI: 10.1107/S2056989017016590/xi2002sup1.cif

Structure factors: contains datablock(s) I. DOI: 10.1107/S2056989017016590/xi2002Isup2.hkl

Supporting information file. DOI: 10.1107/S2056989017016590/xi2002Isup3.cml

CCDC reference: 1578858

Additional supporting information:  crystallographic information; 3D view; checkCIF report

## Figures and Tables

**Table 1:** Hydrogen-bond geometry (Å, °)

*D*—H⋯*A*	*D*—H	H⋯*A*	*D*⋯*A*	*D*—H⋯*A*
C6—H6⋯F1	0.95	2.48	3.004 (2)	115
C6—H6⋯F3	0.95	2.55	3.088 (2)	116
C5—H5⋯F2^i^	0.95	2.63	3.522 (2)	156
C4—H4⋯O1^i^	0.95	2.74	3.490 (2)	136
C6—H6⋯F2^ii^	0.95	2.69	3.614 (2)	163
C6—H6⋯F3^ii^	0.95	2.94	3.584 (2)	126
C5—H5⋯F3^ii^	0.95	2.98	3.603 (2)	124
C3—H3⋯O1^iii^	0.95	2.95	3.882 (2)	166

Symmetry codes: (i) 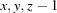; (ii) 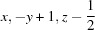; (iii) 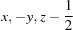.

**Table 2:** Stabilization energies (in kJ mol^−1^) of the individual mol­ecular pairs CD = centroid–centroid distance.

Motif	Symmetry	CD (Å)	*E*_Coul_	*E*_Pol_	*E*_Disp_	*E*_Rep_	*E*_Tot_	Possible Inter­actions	Geometry (Å, °)
I	−*x* + 1, *y*, −*z* + 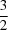	3.731	−5.6	−1.7	−26.2	14.6	−18.8	C7⋯C6	3.6668 (1)
								C1⋯C1	3.6035 (1)
								C2⋯C2	3.5545 (1)
								C8—F3⋯F3—C8	2.8743 (1), 139, 139
II	−*x* + 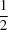 , −*y* + 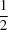 , −*z* + 1	5.470	−3.5	−0.9	−20.4	10.2	−14.5	π–π stacking	3.7869 (1)
								C8—F1⋯C4	3.2425 (1), 134
III	−*x* + 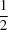 , −*y* + 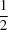 , −*z* + 2	5.274	−5.2	−1.5	−12.6	6.7	−12.7	C7—O1⋯F2—C8	3.1436 (1), 100, 96
								C7—O1⋯F1—C8	3.0457, 139, 90
IV	*x*, *y*, *z* + 1	8.360	−6.4	−1.6	−6.8	4.8	−10.0	C4—H4⋯O1	2.75, 134
								C5—H5⋯F2	2.63, 154
V	*x*, −*y*, *z* + 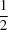	8.524	−1.3	−2.3	−10.0	6.8	−6.9	H3⋯H2	2.40
								C3—H3⋯O1	2.95, 165
VI	*x*, −*y* + 1, *z* + 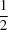	6.652	−0.7	−0.8	−8.2	3.7	−6.0	C6—H6⋯F2	2.69, 163
								C6—H6⋯F3	2.94, 124
								C8—F1⋯F2—C8	3.1023, 114, 147

**Table 3:** Experimental details

Crystal data	
Chemical formula	C_8_H_5_F_3_O
*M*_r_	174.12
Crystal system, space group	Monoclinic, *C*2/*c*
Temperature (K)	110
*a*, *b*, *c* (Å)	13.8129 (3), 12.6034 (2), 8.3595 (2)
β (°)	90.396 (1)
*V* (Å^3^)	1455.27 (5)
*Z*	8
Radiation type	Mo *K*α
μ (mm^−1^)	0.16
Crystal size (mm)	0.30 × 0.30 × 0.30

Data collection	
Diffractometer	Bruker APEXII CCD
Absorption correction	Multi-scan (*SADABS*; Bruker, 2012 ▸)
*T*_min_, *T*_max_	0.697, 0.746
No. of measured, independent and observed [*I* > 2σ(*I*)] reflections	9958, 1045, 944
*R*_int_	0.014
(sin θ/λ)_max_ (Å^−1^)	0.631

Refinement	
*R*[*F* ^2^ > 2σ(*F* ^2^)], *wR*(*F* ^2^), *S*	0.024, 0.064, 1.08
No. of reflections	1045
No. of parameters	109
H-atom treatment	H-atom parameters constrained
Δρ_max_, Δρ_min_ (e Å^−3^)	0.19, −0.20

Computer programs: *APEX2* and *SAINT* (Bruker, 2012 ▸), *SIR92* (Altomare *et al.*, 1994 ▸), *SHELXL2016/6* (Sheldrick, 2015 ▸), *Mercury* (Macrae *et al.*, 2008 ▸), *CIFTAB* (Sheldrick, 2008 ▸) and *PLATON* (Spek, 2009 ▸).

## supplementary crystallographic information

### Crystal data

**Table:**

C_8_H_5_F_3_O	*F*(000) = 704
*M**_r_* = 174.12	*D*_x_ = 1.589 Mg m^−^^3^
Monoclinic, *C*2/*c*	Mo *K*α radiation, λ = 0.71073 Å
*a* = 13.8129 (3) Å	Cell parameters from 5549 reflections
*b* = 12.6034 (2) Å	θ = 2.2–30.2°
*c* = 8.3595 (2) Å	µ = 0.16 mm^−^^1^
β = 90.396 (1)°	*T* = 110 K
*V* = 1455.27 (5) Å^3^	Block, colorless
*Z* = 8	0.30 × 0.30 × 0.30 mm

### Data collection

**Table:**

Bruker APEXII CCD diffractometer	944 reflections with *I* > 2σ(*I*)
Radiation source: fine focus sealed tube	*R*_int_ = 0.014
ω scans	θ_max_ = 26.7°, θ_min_ = 2.2°
Absorption correction: multi-scan (SADABS; Bruker, 2008)	*h* = −17→17
*T*_min_ = 0.697, *T*_max_ = 0.746	*k* = −15→15
9958 measured reflections	*l* = −5→5
1045 independent reflections	

### Refinement

**Table:**

Refinement on *F*^2^	0 restraints
Least-squares matrix: full	Hydrogen site location: inferred from neighbouring sites
*R*[*F*^2^ > 2σ(*F*^2^)] = 0.024	H-atom parameters constrained
*wR*(*F*^2^) = 0.064	*w* = 1/[σ^2^(*F*_o_^2^) + (0.0305*P*)^2^ + 0.8314*P*] where *P* = (*F*_o_^2^ + 2*F*_c_^2^)/3
*S* = 1.08	(Δ/σ)_max_ < 0.001
1045 reflections	Δρ_max_ = 0.19 e Å^−^^3^
109 parameters	Δρ_min_ = −0.20 e Å^−^^3^

### Special details

**Table:**

Geometry. All esds (except the esd in the dihedral angle between two l.s. planes) are estimated using the full covariance matrix. The cell esds are taken into account individually in the estimation of esds in distances, angles and torsion angles; correlations between esds in cell parameters are only used when they are defined by crystal symmetry. An approximate (isotropic) treatment of cell esds is used for estimating esds involving l.s. planes.

### Fractional atomic coordinates and isotropic or equivalent isotropic displacement parameters (Å^2^)

**Table:**

	*x*	*y*	*z*	*U*_iso_*/*U*_eq_	
F1	0.27357 (5)	0.43371 (5)	0.83259 (12)	0.0310 (3)	
F2	0.33321 (6)	0.40770 (6)	1.06711 (16)	0.0376 (4)	
F3	0.42716 (5)	0.44771 (5)	0.87193 (13)	0.0331 (3)	
O1	0.36355 (6)	0.21263 (7)	0.98806 (15)	0.0268 (3)	
C4	0.39488 (8)	0.16370 (10)	0.3960 (2)	0.0278 (5)	
H4	0.402903	0.138083	0.290060	0.033*	
C5	0.38764 (9)	0.27193 (10)	0.4248 (2)	0.0273 (5)	
H5	0.390579	0.320406	0.337898	0.033*	
C6	0.37625 (8)	0.30955 (9)	0.5781 (2)	0.0223 (5)	
H6	0.371252	0.383735	0.596420	0.027*	
C1	0.37202 (7)	0.23921 (9)	0.7068 (2)	0.0176 (5)	
C7	0.36188 (7)	0.27209 (9)	0.8742 (2)	0.0200 (5)	
C8	0.34856 (9)	0.39204 (9)	0.9128 (3)	0.0241 (5)	
C3	0.39028 (9)	0.09306 (9)	0.5237 (2)	0.0259 (5)	
H3	0.395028	0.018927	0.504787	0.031*	
C2	0.37893 (8)	0.12990 (9)	0.6766 (2)	0.0235 (5)	
H2	0.375707	0.081052	0.762999	0.028*	

### Atomic displacement parameters (Å^2^)

**Table:**

	*U*^11^	*U*^22^	*U*^33^	*U*^12^	*U*^13^	*U*^23^
F1	0.0323 (4)	0.0267 (4)	0.0339 (10)	0.0079 (3)	0.0017 (4)	0.0015 (3)
F2	0.0604 (5)	0.0314 (4)	0.0210 (13)	0.0015 (3)	0.0051 (5)	−0.0069 (4)
F3	0.0336 (4)	0.0239 (3)	0.0420 (9)	−0.0065 (3)	0.0030 (4)	−0.0046 (3)
O1	0.0382 (5)	0.0281 (4)	0.0142 (12)	−0.0003 (3)	0.0004 (4)	0.0047 (5)
C4	0.0263 (6)	0.0403 (7)	0.0169 (16)	−0.0026 (5)	0.0001 (6)	−0.0063 (7)
C5	0.0330 (6)	0.0347 (7)	0.0141 (19)	−0.0028 (5)	0.0007 (6)	0.0079 (7)
C6	0.0281 (6)	0.0234 (6)	0.0156 (18)	−0.0001 (4)	0.0004 (6)	0.0031 (6)
C1	0.0193 (5)	0.0217 (5)	0.0118 (17)	−0.0007 (4)	−0.0007 (5)	0.0013 (6)
C7	0.0207 (5)	0.0220 (6)	0.0171 (17)	−0.0011 (4)	−0.0002 (5)	0.0032 (7)
C8	0.0296 (6)	0.0248 (6)	0.018 (2)	−0.0003 (4)	0.0021 (6)	−0.0021 (6)
C3	0.0338 (6)	0.0253 (6)	0.0187 (17)	−0.0013 (4)	0.0001 (6)	−0.0048 (7)
C2	0.0293 (6)	0.0215 (6)	0.0197 (18)	−0.0011 (4)	−0.0012 (6)	0.0026 (6)

### Geometric parameters (Å, º)

**Table:**

F1—C8	1.3373 (17)	C6—C1	1.396 (2)
F2—C8	1.324 (2)	C6—H6	0.9500
F3—C8	1.3389 (15)	C1—C2	1.4040 (15)
O1—C7	1.2112 (18)	C1—C7	1.467 (2)
C4—C5	1.3888 (19)	C7—C8	1.5569 (16)
C4—C3	1.392 (2)	C3—C2	1.370 (2)
C4—H4	0.9500	C3—H3	0.9500
C5—C6	1.377 (2)	C2—H2	0.9500
C5—H5	0.9500		
			
C5—C4—C3	119.46 (17)	C1—C7—C8	118.93 (13)
C5—C4—H4	120.3	F2—C8—F1	107.54 (12)
C3—C4—H4	120.3	F2—C8—F3	107.82 (12)
C6—C5—C4	120.53 (15)	F1—C8—F3	107.05 (12)
C6—C5—H5	119.7	F2—C8—C7	111.48 (12)
C4—C5—H5	119.7	F1—C8—C7	111.71 (12)
C5—C6—C1	120.32 (13)	F3—C8—C7	111.03 (11)
C5—C6—H6	119.8	C2—C3—C4	120.33 (13)
C1—C6—H6	119.8	C2—C3—H3	119.8
C6—C1—C2	118.78 (16)	C4—C3—H3	119.8
C6—C1—C7	124.10 (12)	C3—C2—C1	120.58 (14)
C2—C1—C7	117.11 (13)	C3—C2—H2	119.7
O1—C7—C1	124.96 (12)	C1—C2—H2	119.7
O1—C7—C8	116.10 (16)		
			
C3—C4—C5—C6	0.17 (18)	C1—C7—C8—F2	175.26 (9)
C4—C5—C6—C1	0.12 (17)	O1—C7—C8—F1	−125.49 (14)
C5—C6—C1—C2	−0.42 (16)	C1—C7—C8—F1	54.91 (16)
C5—C6—C1—C7	178.79 (10)	O1—C7—C8—F3	115.10 (15)
C6—C1—C7—O1	−175.62 (11)	C1—C7—C8—F3	−64.50 (17)
C2—C1—C7—O1	3.60 (16)	C5—C4—C3—C2	−0.15 (18)
C6—C1—C7—C8	3.94 (15)	C4—C3—C2—C1	−0.15 (17)
C2—C1—C7—C8	−176.84 (10)	C6—C1—C2—C3	0.43 (16)
O1—C7—C8—F2	−5.15 (14)	C7—C1—C2—C3	−178.83 (10)

### Hydrogen-bond geometry (Å, º)

**Table:**

*D*—H···*A*	*D*—H	H···*A*	*D*···*A*	*D*—H···*A*
C6—H6···F1	0.95	2.48	3.004 (2)	115
C6—H6···F3	0.95	2.55	3.088 (2)	116
C5—H5···F2^i^	0.95	2.63	3.522 (2)	156
C4—H4···O1^i^	0.95	2.74	3.490 (2)	136
C6—H6···F2^ii^	0.95	2.69	3.614 (2)	163
C6—H6···F3^ii^	0.95	2.94	3.584 (2)	126
C5—H5···F3^ii^	0.95	2.98	3.603 (2)	124
C3—H3···O1^iii^	0.95	2.95	3.882 (2)	166

Symmetry codes: (i) *x*, *y*, *z*−1; (ii) *x*, −*y*+1, *z*−1/2; (iii) *x*, −*y*, *z*−1/2.

## References

[bb1] Allen, F. H. (1986). *Acta Cryst.* B**42**, 515–522.

[bb2] Altomare, A., Cascarano, G., Giacovazzo, C., Guagliardi, A., Burla, M. C., Polidori, G. & Camalli, M. (1994). *J. Appl. Cryst.* **27**, 435.

[bb3] Boese, R., Kirchner, M. T., Billups, W. E. & Norman, L. R. (2003). *Angew. Chem. Int. Ed.* **42**, 1961–1963. 10.1002/anie.200250634 12730982

[bb4] Bruker (2012). *APEX2*, *SAINT* and *SADABS*. Bruker AXS Inc., Madison, Wisconsin, USA.

[bb5] Cakl, Z., Reimann, S., Schmidt, E., Moreno, A., Mallat, T. & Baiker, A. (2011). *J. Catal.* **280**, 104–115.

[bb6] Chopra, D., Thiruvenkatam, V., Manjunath, S. G. & Guru Row, T. N. (2007). *Cryst. Growth Des.* **7**, 868–874.

[bb7] Choudhury, A. R., Winterton, N., Steiner, A., Cooper, A. I. & Johnson, K. A. (2005). *J. Am. Chem. Soc.* **127**, 16792–16793. 10.1021/ja055956u 16316218

[bb8] Dey, D., Bhandary, S., Sirohiwal, A., Hathwar, V. R. & Chopra, D. (2016*a*). *Chem. Commun.* **52**, 7225–7228. 10.1039/c6cc02535h 27149236

[bb9] Dey, D., Bhandary, S., Thomas, S. P., Spackman, M. A. & Chopra, D. (2016*b*). *Phys. Chem. Chem. Phys.* **18**, 31811–31820. 10.1039/c6cp05917a 27841399

[bb10] Frisch, M. J., *et al.* (2009). *GAUSSIAN*09, Revision D. 01. Gaussian Inc., Wallingford, CT, USA.

[bb11] Gavezzotti, A. (2003). *J. Phys. Chem. B*, **107**, 2344–2353.

[bb12] Gavezzotti, A. (2011). *New J. Chem.* **35**, 1360–1368.

[bb13] Goubert, G., Demers-Carpentier, V., Masini, F., Dong, Y., Lemay, J. C. & McBreen, P. H. (2011). *Chem. Commun.* **47**, 9113–9115. 10.1039/c1cc12538a 21735010

[bb14] Guzmán-Gutiérrez, M. T., Zolotukhin, M. G., Fritsch, D., Ruiz-Treviño, F. A., Cedillo, G., Fregoso-Israel, E., Ortiz-Estrada, C., Chavez, J. & Kudla, C. (2008). *J. Membr. Sci.* **323**, 379–385.

[bb15] Limnios, D. & Kokotos, C. G. (2014*a*). *J. Org. Chem.* **79**, 4270–4276. 10.1021/jo5003938 24735070

[bb16] Limnios, D. & Kokotos, C. G. (2014*b*). *Chem. Eur. J.* **20**, 559–563. 10.1002/chem.201303360 24302616

[bb17] Macrae, C. F., Bruno, I. J., Chisholm, J. A., Edgington, P. R., McCabe, P., Pidcock, E., Rodriguez-Monge, L., Taylor, R., van de Streek, J. & Wood, P. A. (2008). *J. Appl. Cryst.* **41**, 466–470.

[bb18] McKinnon, J. J., Jayatilaka, D. & Spackman, M. A. (2007). *Chem. Commun.* 3814-3816. 10.1039/b704980c 18217656

[bb19] Sheldrick, G. M. (2008). *Acta Cryst.* A**64**, 112–122. 10.1107/S0108767307043930 18156677

[bb20] Sheldrick, G. M. (2015). *Acta Cryst.* C**71**, 3–8.

[bb21] Sirohiwal, A., Hathwar, V. R., Dey, D. & Chopra, D. (2017*a*). *ChemPhysChem*, **18**, 2859–2863. 10.1002/cphc.201700585 28766864

[bb22] Sirohiwal, A., Hathwar, V. R., Dey, D., Regunathan, R. & Chopra, D. (2017*b*). *Acta Cryst.* B**73**, 140–152. 10.1107/S2052520616017492 28362276

[bb23] Spackman, M. A. & McKinnon, J. J. (2002). *CrystEngComm*, **4**, 378–392.

[bb24] Spek, A. L. (2009). *Acta Cryst.* D**65**, 148–155. 10.1107/S090744490804362X PMC2631630 19171970

[bb25] Theodorou, A. & Kokotos, C. G. (2017*a*). *Green Chem.* **19**, 670–674.

[bb26] Theodorou, A. & Kokotos, C. G. (2017*b*). *Adv. Synth. Catal.* **359**, 1577–1581.

[bb27] Triandafillidi, I. & Kokotos, C. G. (2017). *Org. Lett.* **19**, 106–109. 10.1021/acs.orglett.6b03380 28029257

[bb28] Turner, M. J., McKinnon, J. J., Wolff, S. K., Grimwood, D. J., Spackman, P. R., Jayatilaka, D. & Spackman, M. A. (2017). *CrystalExplorer17*, University of Western Australia.http://hirshfeldsurface.net

